# Rosemary Extracts Upregulate Nrf2, Sestrin2, and MRP2 Protein Level in Human Hepatoma HepG2 Cells

**DOI:** 10.1155/2017/7359806

**Published:** 2017-02-13

**Authors:** Xiao-pei Tong, Yan-xia Ma, Dan-ni Quan, Ling Zhang, Miao Yan, Xin-rong Fan

**Affiliations:** ^1^Department of Pharmacy, The Second Xiangya Hospital, Central South University, Changsha, Hunan Province 410011, China; ^2^Hunan University of Chinese Medicine, Changsha, Hunan Province 410208, China; ^3^China Academy of Chinese Medical Sciences, Beijing 100700, China

## Abstract

In the past few decades, the incidence of liver cancer has been rapidly rising across the world. Rosemary is known to possess antioxidant activity and is used as natural antioxidant food preservative. It is proposed to have anticancer activity in treating different tumor models. In this study, we try to explore the impact of rosemary extracts on upregulating the level of Nrf2 and Nrf2-regulatory proteins, Sestrin2 and MRP2 in HepG2 cells, and to speculate its potential mechanism. The anticancer activity of rosemary extract, including its polyphenolic diterpenes carnosic acid and carnosol, was evaluated to understand the potential effect on HepG2 cells. Rosemary extract, carnosic acid, and carnosol induced the expression of Sestrin2 and MRP2 associate with enhancement of Nrf2 protein level in HepG2 cells, in which carnosic acid showed most obvious effect. Although the activation pathway of Nrf2/ARE was not exactly assessed, it can be assumed that the enhancement of expression of Sestrin2 and MRP2 may result from upregulation of Nrf2.

## 1. Introduction

Nrf2/ARE signaling pathway plays significant role in protecting cells from acute and chronic cell injury. Nrf2 regulates the expression of phase II antioxidant enzymes genes by controlling the antioxidant response element (ARE) sequences. Nrf2/ARE pathway is considered as a new way in mediating the antioxidant response in cancer and other chronic degenerative diseases linked to oxidative stress [1, 2].

Many naturally occurring compounds have been shown to have a wide range of pharmacological properties, which can have potent cancer chemopreventive properties, similar to beta-naphtoflavone, tert-butyl hydroquinone, and phenethyl isothiocyanate [3].

Rosemary* (Rosmarinus officinalis)* is an aromatic evergreen herb native to the Mediterranean region, which is an important component of the Mediterranean diet, and has been used in Traditional Chines medicine. Modern pharmacology has demonstrated that rosemary has functions of antiatherogenic [4], antidepressant [5, 6], antioxidant, and anti- inflammatory [7]. Rosemary extracts, especially its diterpenes (e.g., carnosic acid and carnosol) have been proposed to have anticancer activities in treating different tumor models [1, 2, 8]. Rosemary has diverse actions including activation in cell signaling or cell cycle dynamics in hepatoma cells, against oxidative injury through the SIRT1 pathway, and autophagic cell death induction through inhibition of the Akt/mTOR pathway.

Extensively, rosemary can also act as anticancer agent in cell system, which can conduct cytoprotective effects. This hypothesis is based on its antioxidant activity. As Nrf2/ARE pathway is significant cytoprotective medium in cell system, we evaluated the protein level of Nrf2 and Nrf2-regulatory proteins, such as Sestrin2 and MRP2 on HepG2 cells, treated by different concentrations of rosemary extracts to investigate its chemoprevention and anticancer effect on HepG2 cells.

## 2. Materials and Methods

### 2.1. Rosemary and Its Compounds, Kits, and Antibodies

Rosemary extract (RE, contained carnosic acid 23.2%, carnosol 12.4%), carnosic acid (CA, above 99% purity), and carnosol (CL, above 99% purity) were obtained from OnRoad Biotech Co. (Changsha, China). MTT [3-(4, 5-dimethylthiazol-2-yl)-2, 5-diphenyltetrazolium bromide] was from THEMO cleaved caspase-3. ELISA kit was obtained from Cusabio Biotech Co. Ltd. (Wuhan, China). Antibodies for Western Blot: anti-Nrf2 antibody (ab137550) and anti-MRP2 antibody (ab172630) were from Abcam (USA). Sestrin2 (H-62) antibody was from Santa Cruz Biotechnology, Inc. (Santa Cruz, CA, USA). BCA protein assay kit was obtained from Beijing ComWin Biotech Co., Ltd. (Beijing, China). One-Step RT-PCR kit was obtained from Life Technologies (Grand Island, NY, USA). RNeasy mini kit and RNase-Free DNase set for RNA extraction were obtained from QIAGEN (Santa Clarita, CA, USA).

### 2.2. Cell Culture and Treatment

Human hepatoma cell line (HepG2) was obtained from Xiangya Cell Bank (Central South University, Changsha, China), cultured in Dulbecco modified eagle medium (Hyclone, Logan, USA) supplemented with 10% fetal bovine serum (Sijiqing, Hangzhou, China) and 1% penicillin/streptomycin in a 37°C incubator with 5% CO_2_. Cells were cultured in mediums supplemented with a range of concentrations of RE, CA, or CL for designed times. RE, CA, and CL were solubilized and delivered in dimethyl sulfoxide (DMSO) and stored at the temperature of −20°C. Drugs were freshly diluted to the indicated concentrations with culture medium before use. The final DMSO concentration in the media for RE was 1% and for CA or CL was 0.1%, respectively. Vehicle controls were employed in all experiments. Treatments started after cells attached for 24 hours.

### 2.3. Cell Viability (MTT Assay)

Cell viability was measured by 3-(4, 5-dimethylthiazol-2-yl)-2, 5-diphenyltetrazolium bromide (MTT) reduction assay. HepG2 cells seeding density was about 2 × 10^4^ in each well. Cells were cultured in mediums which were supplemented with a range of concentrations of RE (0, 10, 20, 30, 40, 50, 75, and 100 *μ*g/mL) or CA and CL (0, 10, 20, 30, 40, 50, 75, and 100 *μ*M), respectively, for 24, 48, or 72 hours. The concentration gradients choice was based on the reference [9]. Then MTT (5 mg/mL) was added to each well (300 *μ*L) and incubated for 3–5 hours, after which the MTT was removed and DMSO (300 *μ*L) was added to each well. After shaking for 10 minutes, 100 *μ*L of each sample was transferred to a 96-well microtiter plate and the absorbance was recorded at 540 nm.

### 2.4. ELISA

Cleaved caspase-3 levels in same amounts of protein in different cell lysates were detected with ELISA. All procedures were conducted according to the manufacturer's manual (Huamei, Wuhan, China). The seeding density of HepG2 cells was 1 × 10^6^, respectively, in each 10 cm^2^ plate. Cells were cultured in mediums which were supplemented with a range of concentrations of RE (0, 30, 50, and 100 *μ*g/mL) or CA and CL (0, 30, 50, and 100 *μ*M), respectively, for 24 hours.

### 2.5. Western Blots

Whole cell lysates from treated cells were prepared as previously described. Lysates were quantified for protein concentration by using BCA assay according to the manufacturer's manual. A common Western blots protocol was followed [10]. Briefly, similar amounts of protein were loaded to each well of 8% or 10% precast gels (Bio-Rad, Hercules, CA, USA). After transfer, membranes were blocked with 5% skim milk powder solution and incubated with primary antibody (1 : 1000 or 1 : 2000) overnight at the temperature of 4°C, rinsed briefly, and then incubated with secondary antibody (1 : 10000) at room temperature for 1 hour. Membranes were washed, incubated with substrates, and exposed in a FluorChem E imager (Bio-Rad, Hercules, CA, USA). The seeding densities of HepG2 cells were 1 × 10^6^, respectively, in each 10 cm^2^ plate. Cells were cultured medium supplemented with a range of concentrations of RE (0, 30, 50, and 100 *μ*g/mL) or CA and CL (0, 30, 50, and 100 *μ*M), respectively, for 24 hours. Western blots were performed with 3 separate lysates at least. Separate lysates were analyzed 3 times by Western Blot to confirm the results.

### 2.6. RT-PCR

(See Table 1). Primers of Sestrin2 and RT-PCR were synthesized by IDT (Integrated DNA Technologies) (Coralville, IA, USA). HepG2 cells were treated with 40 *μ*g/mL RE or 60 *μ*M CA for 24 hours. Total RNA was extracted from treated and control cells. One microgram of total RNA was used as template for one-step RT-PCR. The manufacturer's protocol was followed. The seeding densities of HepG2 cells were 1 × 10^6^ in each 10 cm^2^ plate. Cells were cultured in medium supplemented with a range of concentrations of RE (0, 30, 50, and 100 *μ*g/mL) or CA and CL (0, 30, 50, and 100 *μ*M), respectively, for 24 hours.

### 2.7. Statistical Analysis

Results of the experiment were reported as mean ± standard deviation (SD) and conducted with SPSS19.0. All data were analyzed by one-way ANOVA, tested by Tukey's test for multiple comparisons. All statistical tests were two-sided. *P* < 0.05 was considered statistically significant.

## 3. Results

### 3.1. RE, CA, and CL Reduced Hepatoma Cell Viability

**Figure 1:**
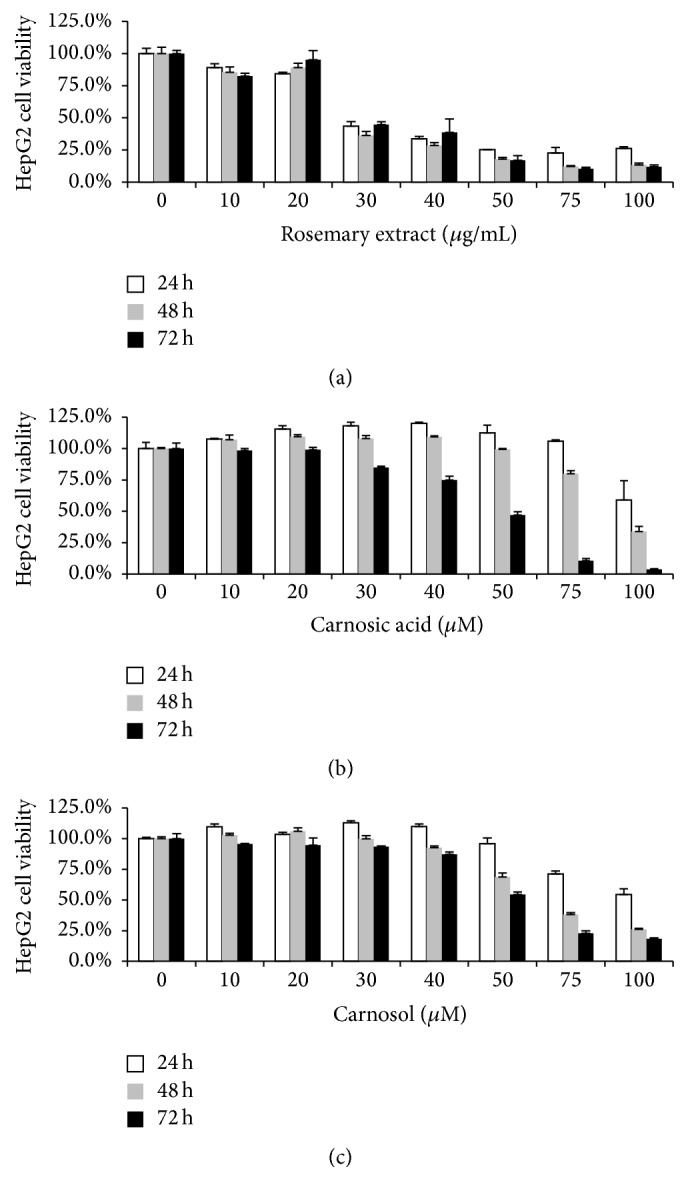
Rosemary extract (RE), carnosic acid (CA), and carnosol (CL) decreased cell viability on HepG2 cells. HepG2 cells (2 × 10^4^cells per well) were treated with various concentrations of RE (a) (0, 10, 20, 30, 40, 50, 75, and 100 *μ*g/mL), CA (b) (0, 10, 20, 30, 40, 50, 75, and 100 *μ*M), and CL (c) (0, 10, 20, 30, 40, 50, 75, and 100 *μ*M) for 24, 48, and 72 hrs. Results are represented by statistical mean along with standard deviation.

The impacts of RE, CA, and CL on HepG2 cell viability were determined. HepG2 cells were treated with RE (0, 10, 20, 30, 40, 50, 75, and 100 *μ*g/mL) or CA and CL (0, 10, 20, 30, 40, 50, 75, and 100 *μ*M) for 24, 48, and 72 hours, respectively. MTT results showed that RE, CA, and CL decreased HepG2 cell viability in dose and time-dependent manner (Figure 1). Among them, carnosic acid had the most obvious effect.

### 3.2. RE, CA, and CL Increased the Degree of Caspase-3 Level in Hepatoma Cancer Cells

**Figure 2:**
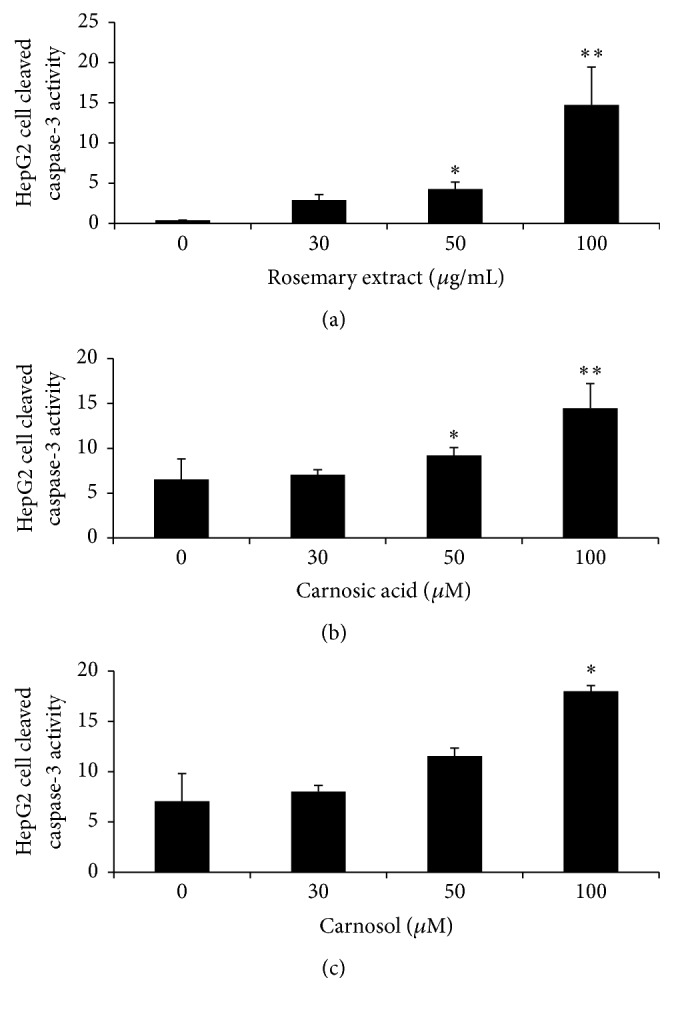
Rosemary extract (RE), carnosic acid (CA), and carnosol (CL) increased the degree of caspase-3 levels in HepG2 cells. HepG2 cells (1 × 10^6^ cells per well) were treated with RE (0, 30, 50, and 100 *μ*g/mL) or CA and CL (0, 30, 50, and 100 *μ*M), respectively, for 24 hrs. (a) Cleaved caspase-3 level in RE treated HepG2 cells after 24 hrs. (b) Cleaved caspase-3 level in CA treated HepG2 cells after 24 hrs. (c) Cleaved caspase-3 level in CL treated HepG2 cells after 24 hrs. Results are represented by statistical mean along with standard deviation, ^*∗*^*P* < 0.05 and ^*∗∗*^*P* < 0.01.

Whole cell lysates from HepG2 cells were subjected to ELISA assays of cleaved caspase-3 by using ELISA kit. BCA kit was used to establish standard curve to measure the protein level. ELISA data revealed that the degree of caspase-3 levels in HepG2 cells treated with RE (0, 30, 50, and 100 *µ*g/mL) and CA and CL (0, 30, 50, and 100 *µ*M) was increased in a dose-dependent manner after being treated for 24 hours (Figure 2). Rosemary extract significantly increased cleaved caspase-3 at 50 *µ*g/mL (*P* < 0.05) and 100 *µ*g/mL (*P* < 0.01) after 24 hours. Significant increase of cleavage of caspase-3 from carnosic acid was also observed at 50 *µ*M (*P* < 0.05) and 100 *µ*M (*P* < 0.01) after 24 hours, while carnosol increased cleaved caspase-3 after 24 hours at 100 *µ*M (*P* < 0.05).

### 3.3. RE, CA, and CL Increased Expression of Sestrin2 and MRP2 Concomitantly with Enhanced Nrf2 mRNA and Protein Levels in HepG2 Cells

**Figure 3:**
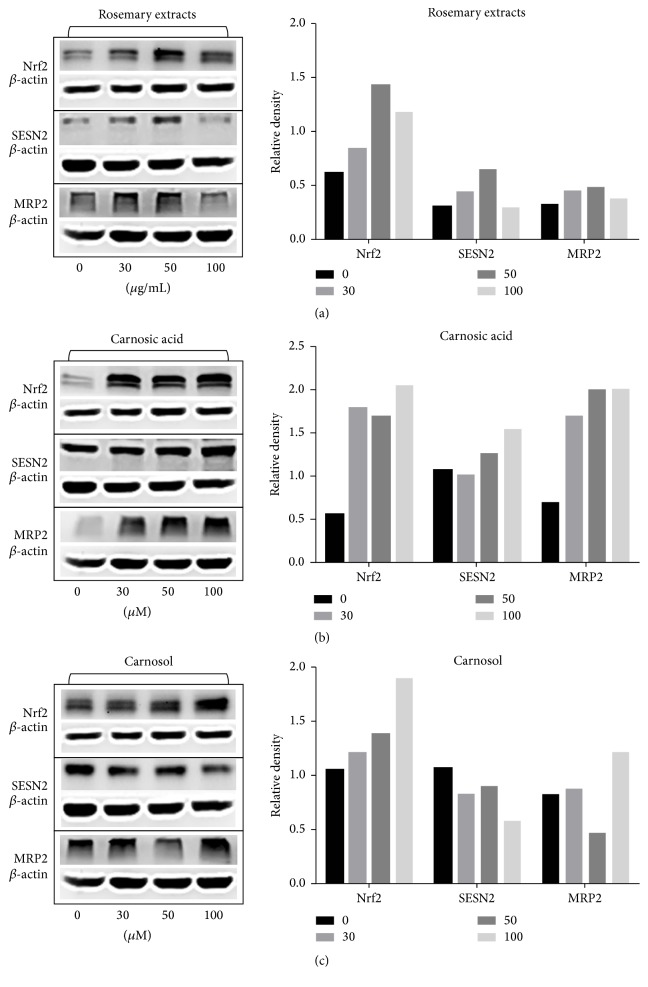
Rosemary extract (RE), carnosic acid (CA), and carnosol (CL) increased the protein levels of Nrf2, Sestrin2, and MRP2 in HepG2 cells. HepG2 cells (1 × 10^6^ cells per well) were treated with RE (0, 30, 50, and 100 *μ*g/mL) or CA and CL (0, 30, 50, and 100 *μ*M), respectively, for 24 hrs. (a) Western Blot analysis of the protein level of Nrf2, MRP2, and Sestrin2 in RE treated HepG2 cells. Left column: protein level in RE treated HepG2 cells after 24 hrs; right column: densitometric analysis of RE Western Blot results. (b) Western Blot analysis of the protein level of Nrf2, MRP2, and Sestrin2 in CA treated HepG2 cells. Left column: protein level in CA treated HepG2 cells after 24 hrs; right column: densitometric analysis of CA Western Blot results. (c) Western Blot analysis of the protein level of Nrf2, MRP2, and Sestrin2 in CL treated HepG2 cells. Left column: protein level in CL treated HepG2 cells after 24 hrs; right column: densitometric analysis of CL Western Blot results.

**Figure 4:**
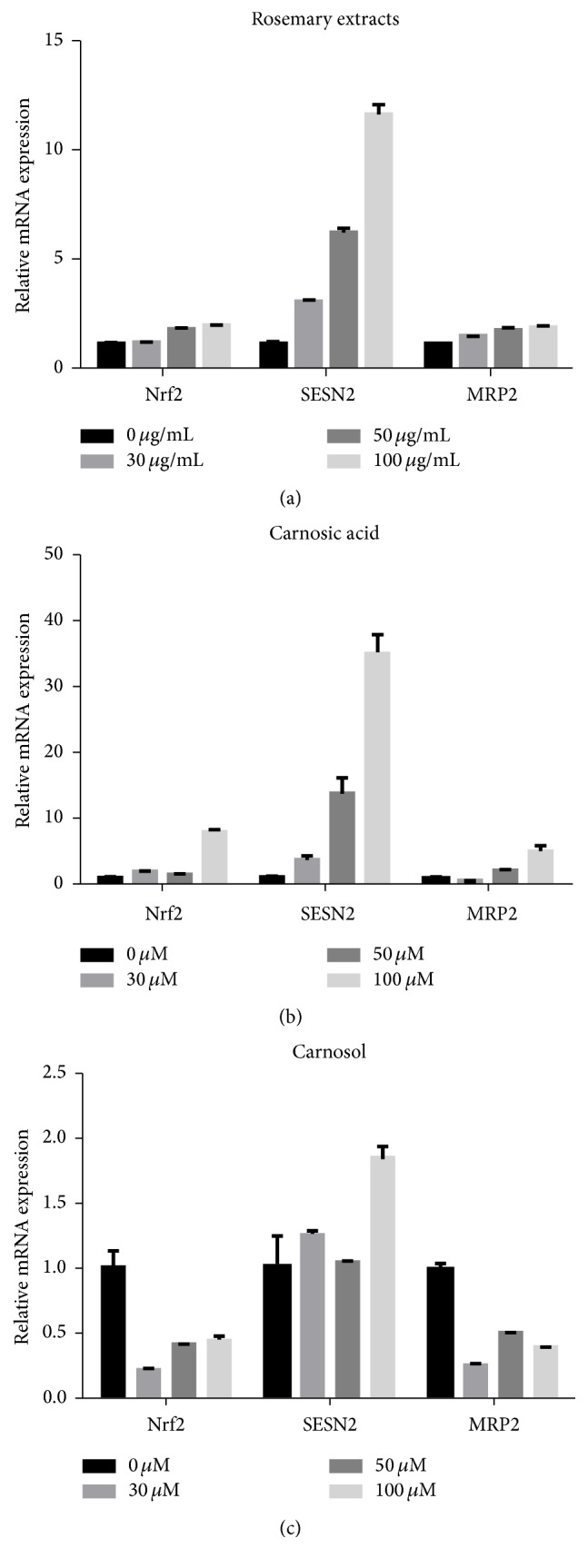
RT-PCR analysis of the mRNA level of Nrf2, MRP2, and Sestrin2 in RE, CA, and CL treated HepG2 cells. (a) mRNA level in RE treated HepG2 cells after 24 hrs; (b) mRNA level in CA treated HepG2 cells after 24 hrs; (c) mRNA level in CL treated HepG2 cells after 24 hrs.

In this part, we detected the protein level and mRNA level of Nrf2, Sestrin2, and MRP2 by Western Blot and RT-PCR, respectively, in HepG2 cells. HepG2 cells were treated with RE (0, 30, 50, and 100 *µ*g/mL) and CA and CL (0, 30, 50, and 100 *µ*M) for 24 hours. Western Blot results showed that the protein level of Nrf2 in HepG2 cells was increased by rosemary extract and carnosol in a dose-dependent manner, while Sestrin2 and MRP2 were inconspicuous. Carnosic acid increased the protein level of Nrf2 accompanied with remarkable increase of Sestrin2 and MRP2 (Figure 3). To interpret the results more directly, we also provided the densitometric analysis to make quantitative determination of Western Blot (Figure 3). RT-PCR results showed that the effects of RE, CA, and CL on mRNA were generally consistent with the protein done (Figure 4).

## 4. Discussion and Conclusion

Rosemary and its polyphenolic diterpene, carnosic acid and carnosol, are known to possess antioxidant activity and are used as natural antioxidant food preservative widely. The most abundant polyphenolic diterpene of rosemary is carnosic acid. It has been thought to be the primary contributor for antioxidant activity [10]. Compared to its antioxidant activity, the anticancer and chemoprevention effects were more intrigued to us.

Sestrin2 is a kind of Nrf2-regulatory protein that may function in the regulation of cell growth and survival and be involved in protecting against oxidative stress. It may be involved in cellular response in different stress conditions [11]. The increase of the expression of Sestrin2 is thought to be cytoprotective. MRP2 is one of the multidrug resistance-associated proteins, which is also regulated by Nrf2. It is thought as a major impediment to the success of cancer chemotherapy. There are many literatures reported that MPRs lead to drug resistance or drug-drug interactions in the process of drug treatment [12]. Normally, MPR2 can also function on cellular detoxification, oxidative stress, inflammation, material transportation, and so on. Chemoprophylactic activity of MRP2 is based on the role of efflux transporter. It can excrete xenobiotics and resist the damage when cells were exposed to external stimulus. The increase of the expression of MRP2 is thought to be chemoprevention. In this study, we detected the protein level of Sestrin2 and MRP2. The results showed that the expression of Sestrin2 and MRP2 was increased after being treated by rosemary extracts. This consequence indirectly probed that the rosemary extracts may possess chemoprevention and anticancer effect on HepG2 cells.

Nrf2/ARE signaling pathway has emerged as novel target for the cancer prevention because of its cytoprotective function. It regulates the expression of phase II antioxidant enzymes genes by controlling the antioxidant response element (ARE) sequences. Sestrin2 and MRP2 are two of the functional proteins regulated by Nrf2. Experimental results show that the expression of Sestrin2 and MRP2 was increased concomitantly with the enhancement of Nrf2 protein levels in HepG2 cells. Although the activation of Nrf2/ARE pathway was not assessed it may be assumed that enhanced expression of Sestrin2 and MRP2 may result from Nrf2 upregulation.

## Figures and Tables

**Table 1:** The sequences and Genbank accession number of Nrf2, MRP2, and Sestrin2.

mRNA	Sequences	Genbank accession number	Size
Actin-F	CGTGGACATCCGCAAAGAC	XM_006715764.1	234 bp
Actin-R	TCGTCATACTCCTGCTTGCTG		
NRF2-F	AACCAGTGGATCTGCCAACTACTC	NM_001145413.2	90 bp
NRF2-R	CTGCGCCAAAAGCTGCAT		
MRP2-F	TGAGCAAGTTTGAAACGCACAT	NM_000392.4	78 bp
MRP2-R	AGCTCTTCTCCTGCCGTCTCT		
SESN2-F	GAGAAGACCACCCGAAGAATGT	NM_031459.4	153 bp
SESN2-R	CAGGAGTCAGGTCATGTAGCGG		

## References

[1] Barni M. V., Carlini M. J., Cafferata E. G., Puricelli L., Moreno S. (2012). Carnosic acid inhibits the proliferation and migration capacity of human colorectal cancer cells. *Oncology Reports*.

[2] Cheng A.-C., Lee M.-F., Tsai M.-L. (2011). Rosmanol potently induces apoptosis through both the mitochondrial apoptotic pathway and death receptor pathway in human colon adenocarcinoma COLO 205 cells. *Food and Chemical Toxicology*.

[3] Krajka-Kuźniak V., Paluszczak J., Szaefer H., Baer-Dubowska W. (2015). The activation of the Nrf2/ARE pathway in HepG2 hepatoma cells by phytochemicals and subsequent modulation of phase II and antioxidant enzyme expression. *Journal of Physiology and Biochemistry*.

[4] Ullevig S. L., Zhao Q., Zamora D., Asmis R. (2011). Ursolic acid protects diabetic mice against monocyte dysfunction and accelerated atherosclerosis. *Atherosclerosis*.

[5] Sasaki K., El Omri A., Kondo S., Han J., Isoda H. (2013). *Rosmarinus officinalis* polyphenols produce anti-depressant like effect through monoaminergic and cholinergic functions modulation. *Behavioural Brain Research*.

[6] Ferlemi A.-V., Katsikoudi A., Kontogianni V. G. (2015). Rosemary tea consumption results to anxiolytic- and anti-depressant-like behavior of adult male mice and inhibits all cerebral area and liver cholinesterase activity; phytochemical investigation and in silico studies. *Chemico-Biological Interactions*.

[7] Bozin B., Mimica-Dukic N., Samojlik I., Jovin E. (2007). Antimicrobial and antioxidant properties of Rosemary and Sage (Rosmarinus officinalis L. and Salvia officinalis L., Lamiaceae) essential oils. *Journal of Agricultural and Food Chemistry*.

[8] Yesil-Celiktas O., Sevimli C., Bedir E., Vardar-Sukan F. (2010). Inhibitory effects of rosemary extracts, carnosic acid and rosmarinic acid on the growth of various human cancer cell lines. *Plant Foods for Human Nutrition*.

[9] Yan M., Li G., Petiwala S. M., Householter E., Johnson J. J. (2015). Standardized rosemary (Rosmarinus officinalis) extract induces Nrf2/sestrin-2 pathway in colon cancer cells. *Journal of Functional Foods*.

[10] Petiwala S. M., Puthenveetil A. G., Johnson J. J. (2013). Polyphenols from the Mediterranean herb rosemary (*Rosmarinus officinalis*) for prostate cancer. *Frontiers in Pharmacology*.

[11] Petiwala S. M., Berhe S., Li G. (2014). Rosemary (Rosmarinus officinalis) extract modulates CHOP/GADD153 to promote androgen receptor degradation and decreases xenograft tumor growth. *PLoS ONE*.

[12] Januchowski R., Sterzyńska K., Zaorska K. (2016). Analysis of MDR genes expression and cross-resistance in eight drug resistant ovarian cancer cell lines. *Journal of Ovarian Research*.

